# Electrochemical Evaluation of Polymer-Based Microelectrode Arrays: Analytical Performance on Oxygen and Hydrogen Peroxide

**DOI:** 10.3390/s26154929

**Published:** 2026-08-04

**Authors:** Eliana Fernandes, Ana Ledo, Kee Scholten, Ellis Meng, Greg A. Gerhardt, Rui M. Barbosa

**Affiliations:** 1 Faculty of Pharmacy, University of Coimbra, Health Science Campus, Azinhaga de Santa Comba, 3000-548 Coimbra, Portugal; eliana.fernandes@ff.uc.pt (E.F.); analedo@ff.uc.pt (A.L.); 2 CNC-UC—Centre for Neuroscience and Cell Biology, CIBB—Centre for Innovative Biomedicine and Biotechnology, University of Coimbra, Edifício FMUC, Rua Larga, 3004-504 Coimbra, Portugal; 3 Alfred E. Mann Department of Biomedical Engineering, University of Southern California, 1042 Downey Way, Denney Research Center (DRB) 140, Los Angeles, CA 90089-1111, USA; kscholte@usc.edu (K.S.); ellismen@usc.edu (E.M.); 4 Centre for Microelectrode Technology (CenMeT), Department of Neuroscience, University of Kentucky Medical Center, MN208, Medical Science Building, 800 Rose Street, Lexington, KY 40536-0298, USA; gregg@uky.edu; 5 LEC—Laboratory for Electroanalysis and Corrosion, IPN—Instituto Pedro Nunes, Rua Pedro Nunes, 3030-199 Coimbra, Portugal

**Keywords:** polymer microelectrode arrays, electrochemical characterization, oxygen reduction, hydrogen peroxide detection

## Abstract

This study investigates the electrochemical properties of polymer-based microelectrode arrays (pMEAs) and their performance in measuring oxygen (O_2_) and hydrogen peroxide (H_2_O_2_). Morphological characterization by scanning electron microscopy (SEM), energy-dispersive X-ray spectroscopy (EDS) and X-ray diffraction (XRD) revealed a uniform, fine-grained platinum surface with nanoscale roughness, consistent with the Ti/Pt/Au/Pt multilayer stack architecture. The electrochemical behavior of the pMEAs was assessed using cyclic voltammetry (CV) and electrochemical impedance spectroscopy (EIS), which demonstrated favorable responses for both O_2_ reduction and H_2_O_2_ oxidation, together with low impedance (41.1 kΩ at 1 kHz). For O_2_ detection, amperometric measurements at −0.6 V vs. Ag/AgCl indicated a sensitivity of −0.25 ± 0.04 nA μM^−1^ and a detection limit of 5.4 ± 1.4 nM. For H_2_O_2_ detection, application of +0.7 V vs. Ag/AgCl resulted in a sensitivity of 88.13 ± 7.61 nA mM^−1^ and a detection limit of 41.9 ± 5.6 nM. Selectivity evaluation showed effective interferent exclusion following *m*-phenylenediamine electrodeposition, without compromising analytical performance. Overall, these findings indicate the suitability of pMEAs for real-time, in vivo monitoring of O_2_ and H_2_O_2_ in brain tissue with high spatial and temporal resolution, supporting applications in oxidative stress research and neurometabolic sensing.

## 1. Introduction

Understanding the intricate relationship between brain chemistry and electrophysiology is fundamental in deciphering how the brain functions, communicates, and maintains homeostasis. This interplay governs how chemical signals, such as neurotransmitters, modulate electrical activity, including action potentials, thereby ensuring efficient neural communication [1,2]. Advancements in electrophysiological and electrochemical sensors and biosensors now enable simultaneous recording of electrochemical and electrophysiological signals, offering a more comprehensive view of neuronal activity and chemical signaling [3,4,5,6,7]. These neural tools are essential for capturing the dynamic interactions between neurotransmitter release, neurometabolic processes, and electrical activity, providing critical insights into both normal brain function and neurological disorders [6,7,8,9].

Microelectrode arrays (MEAs) can be fabricated from diverse substrates, including silicon, ceramics and polymers, and have gained significant relevance in neurobiology both as recording and stimulation probes [10,11,12]. Ceramic-based microelectrodes are widely valued for their mechanical stability, biocompatibility, and excellent electrical properties [11,13,14]. Their robustness allows them to withstand mechanical stresses associated with in vivo applications, while their chemical inertness and established biocompatibility support their use in chronic neural interfacing applications [13,15]. Additionally, these electrodes can be engineered to exhibit favorable electrical characteristics, such as low impedance and high charge injection capacity, which are essential for long-term neural interfacing [16,17]. Further surface modifications using conducting polymers, nanotubes, or nanoparticles can significantly enhance their electrochemical performance, which can improve sensitivity for neurotransmitter detection [18,19].

Polymer-based microelectrodes offer distinct advantages in terms of flexibility, ease of fabrication, and adaptability [20,21]. Their compliant mechanical properties tend to reduce tissue damage during implantation, while their customizable designs allow for tailored electrode configurations [22]. Those fabricated from Parylene C have gained prominence due to their biocompatibility, flexibility and chemical inertness, making them well-suited substrates for long-term neural recording and stimulation [23,24,25,26,27]. The unique properties of Parylene C ensure that the electrodes maintain their electrical characteristics even under bending or other mechanical stress, thereby preserving stability and reliability during in vivo recordings [28].

Despite the growing interest in pMEAs for neural interfacing, the electrochemical properties of Parylene C-based devices featuring multilayer metal stacks have not been systematically characterized to the depth required for their reliable deployment as neurochemical biosensors. In this study, we address this gap by providing a comprehensive electrochemical characterization of a Parylene C-based pMEA (Polymer Implantable Electrode (PIE) Foundry, University of Southern California, CA, USA) with a Ti/Pt/Au/Pt multilayer active surface [26], encompassing surface morphology, electrochemical active surface area determination, impedance spectroscopy, and amperometric detection of O_2_ and H_2_O_2_. We further demonstrate the platform’s compatibility with permselective surface modifications strategies and establish key structure–performance relationships governing electrode sensitivity and selectivity. These results provide the electrochemical foundation necessary for the translation of this flexible polymer platform into functional biosensors for real-time, multimodal neurochemical and electrochemical monitoring in biological systems.

## 2. Materials and Methods

### 2.1. Chemicals and Solutions

All reagents used were of analytical grade and obtained from Sigma-Aldrich, Lisbon, Portugal. In vitro electrode evaluations were conducted in either phosphate-buffered saline (PBS Lite) 0.05 M (pH 7.4), composed of 100 mM NaCl, 10 mM NaH_2_PO_4_ and 40 mM Na_2_HPO_4_, or in an acidic electrolyte medium of 0.1 M H_2_SO_4_. For operational stability assessments in a more biologically representative matrix, artificial cerebrospinal fluid (aCSF) was prepared, composed of 147.6 mM NaCl, 3 mM KCl, 1.4 mM CaCl_2_, 0.8 mM MgCl_2_, and 1 mM NaH_2_PO_4_, supplemented with 10 mM glucose and 0.5 mg mL^−1^ bovine serum albumin (BSA). Unless stated otherwise, all solutions were prepared using bi-deionized water with a resistivity of ≥18 MΩ cm (Millipore Corporation, Burlington, MA, USA). When needed, solutions were deoxygenated by purging with nitrogen gas (Air Liquide, Lisbon, Portugal) for a minimum of 15 min. For electrode calibration, oxygen-saturated solutions were prepared by bubbling PBS Lite with 100% O_2_ (Air Liquide, Lisbon, Portugal) for 15 min, achieving an O_2_ concentration of approximately 1.4 mM concentration at 20 °C [29]. The H_2_O_2_ (9.8 mM), ascorbic acid (20 mM) and dopamine (20 mM) stock solutions were prepared freshly each day.

### 2.2. Polymer Microelectrode Arrays

**Figure 1:**
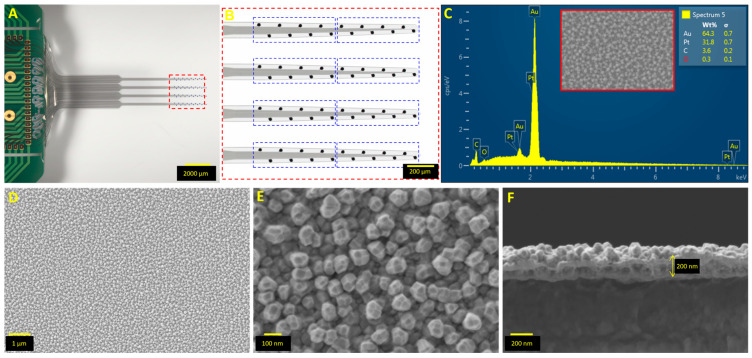
Morphological and chemical analysis of the pMEA Ti/Pt/Au/Pt stack surface. (**A**) Brightfield microscopic image of the MEA and (**B**) high-magnification view of the active surface with the eight zones distinguished by the dashed blue rectangles (region of interest indicated with dashed red square in (**A**)). (**C**) Elemental composition of the Ti/Pt/Au/Pt stack active surface layer of the pMEA obtained by SEM/EDS elemental analysis at 10 keV. (**D**) High-resolution SEM micrograph image of the planar active surface and (**E**) high-magnification view of the Pt layer. (**F**) Cross-sectional SEM micrograph of the stacked metal layer on the silicon carrier wafer, revealing nanoscale elevations on the Pt surface.

In this study, we used a 64-site Parylene C (poly-monochloro (*p*-xylylene))-based polymer microelectrode array, ganged into 8 independent recording zones (8 pads per zone). This array features a multilayered stacked active surface composed of Ti/Pt/Au/Pt layers with thickness of 20/25/155/25 nm, respectively. Each recorded signal captures the aggregate response of up to eight active sites on the electrode by shorting groups of electrodes together. The electrodes in Figure 1 were provided by the PIE Foundry at the University of Southern California, Los Angeles, CA, USA and were previously described in [26]. These pMEAs comprise three key structural components: a base layer of Parylene C, a metal layer containing the electrodes, traces and contact pads, and an insulating top layer of Parylene C. The insulating layer was selectively etched to expose the electrodes and contacts. The fabrication of these pMEAs involves photolithography, chemical vapor and electron beam deposition, and plasma etching, all conducted in a class 1000 (ISO 6) cleanroom environment [26]. Briefly, the fabrication process began with 100 mm diameter silicon carrier wafers, which were vapor-coated in approximately 10 µm of Parylene C (Specialty Coating Systems, Indianapolis, IN, USA) to form the base layer. The wafers were then baked at 150 °C for 4 h under vacuum or a nitrogen atmosphere to increase the crystallinity of the Parylene C, reducing its thermal expansion and preparing it for the subsequent metal deposition.

A thin-film metal layer containing all traces, contact pads, electrodes, and labels was then fabricated using image-reversal photolithography, metal evaporation, and solvent-assisted metal lift-off. The wafers were coated with AZ 5214E-IR photoresist (Integrated Micro Materials, Denton, TX, USA) at a thickness of 1.1 µm and patterned in image-reversal mode. To enhance adhesion, the Parylene C surface was treated with mild O_2_ plasma prior to metal deposition. A Ti/Pt/Au/Pt metal stack (20/25/155/25 nm, respectively) was deposited by e-beam evaporation, and the patterned metal layer was defined via solvent lift-off. Residual unpatterned photoresist was removed in a bath of N-methyl-2-pyrrolidone (NMP) (Integrated Micro Materials, Denton, TX, USA) at 60 °C, followed by ultrasonic treatment.

The wafers were subsequently treated with A174 silane (Specialty Coating Systems, Indianapolis, IN, USA), an adhesion promoter, and coated with a second 10 µm thick Parylene C layer to serve as the insulating top surface of the pMEA, bringing the total multilayer thickness to approximately 20 µm. The electrodes and contact pads were exposed, and the pMEA shape was defined by etching the wafers with O_2_ plasma through two separate photoresist masks. For the first etch, wafers were treated with 15 µm thick AZ 12XT-20PL-15 photoresist (Integrated Micro Materials, Denton, TX, USA), patterned by contact UV lithography and etched to a depth of 10 µm using reactive ion etching. After initial etching step, the photoresist mask was stripped, a second mask was applied, and the etching process was repeated to complete the design. Following the second etch, the mask was stripped and the devices were released by submerging the wafer in deionized water, where individual devices were carefully peeled off using tweezers.

To enhance adhesion between the Parylene C layers and improve barrier properties, the pMEAs were annealed under vacuum at 200 °C for 48 h, sandwiched between ceramic alumina plates (0.635 mm thick), and subsequently at 275 °C for 5 h [26]. Finally, the electrodes were cleaned with a brief O_2_ plasma exposure prior to use.

### 2.3. Scanning Electron Microscopy and Elemental Composition Characterization

High-resolution scanning electron microscopy (SEM) was performed using a Zeiss Merlin scanning electron microscope (Oberkochen, Germany) equipped with a GEMINI II column, offering advanced imaging capabilities with exceptional resolution and contrast. The instrument was coupled with energy-dispersive X-ray spectroscopy (EDS) for elemental analysis, enabling qualitative and quantitative identification of the sample’s composition. EDS analysis was conducted at an accelerating voltage of 10 keV using an X-Max detector (Oxford Instruments, High Wycombe, UK).

### 2.4. X-Ray Diffraction Characterization

The electrode structure was analyzed by X-ray diffraction (XRD) using a Rigaku MiniFlex 600 diffractometer (Rigaku, Tokyo, Japan). Test coupons (approx. 2 × 2 cm^2^) consisting of the stacked metal on a silicon substrate were used. The diffractometer was operated at 40 kV and 15 mA using CuKα radiation (λ = 1.5406 Å), which is well-suited for studying crystalline materials due to its high energy that ensures effective penetration and scattering, and its wavelength, which is ideal for resolving atomic-scale features.

### 2.5. Electrochemical Instrumentation

Electrochemical characterization and amperometric calibrations were performed using a MultiPalmSens4 potentiostat (PalmSens BV, Houten, The Netherlands), controlled by MultiTrace v. 4.2 software. A three-electrode electrochemical cell configuration was employed, with the pMEA as the working electrode, an Ag/AgCl reference electrode (3 M KCl; RE-5B, BASi Research Products Inc., West Lafayette, IN, USA), and a platinum wire as the auxiliary electrode.

### 2.6. Microelectrode Calibration

The pMEAs were evaluated for their response to O_2_ and H_2_O_2_. Amperometric calibrations were conducted in 40 mL of 0.05 M PBS Lite (pH 7.4) at room temperature (20 °C) with continuous stirring at 240 rpm. To remove dissolved O_2_, the calibration solution was purged with N_2_ for a minimum period of 15 min. After purging, the N_2_ delivery needle was repositioned above the solution surface to minimize O_2_ back-diffusion into the calibration medium. Before calibration, the background current was allowed to stabilize under constant polarization for approximately 20 min. Then, 10 µM aliquots of O_2_-saturated solution were sequentially added to obtain a final concentration range of 0–50 µM. To assess the upper limit of the linear range, the O_2_ calibration protocol was extended to a concentration range of 0–150 µM. For the H_2_O_2_ calibrations, once a stable baseline was established, 9.8 µM aliquots of the H_2_O_2_ stock solution were sequentially added to obtain a final concentration range of 0–39.2 µM. To establish the upper limit of the linear range, the H_2_O_2_ calibration protocol described above was extended to a concentration range of 0–2 mM on a subset of electrodes (*n* = 4, same devices/sites as the original calibration). Unless otherwise stated, reported sensitivity, LOD, and calibration parameters (*n* = 8) were obtained from 4 ganged recording sites on each of 2 independently fabricated pMEA devices, capturing both inter-device and inter-site variability.

### 2.7. Electrochemical Impedance Spectroscopy Measurements

Electrochemical impedance spectroscopy (EIS) measurements were performed over a frequency range of 100 kHz to 0.1 Hz (10 frequencies per decade) at the open-circuit potential (OCP = +0.03 V vs. Ag/AgCl). Measurements were carried out in N_2_-saturated 0.05 M PBS Lite (pH 7.4) using a three-electrode configuration. Impedance spectra were fitted to an equivalent circuit comprising the solution resistance (R_s_) in series with a parallel combination of a constant phase element (CPE) and a leakage resistance (R_e_), using complex nonlinear least squares minimization.

### 2.8. Selectivity Measurements

For permselective membrane deposition, *m*-phenylenediamine (*m*-PD) was electropolymerized onto the Pt surface by cyclic voltammetry in deoxygenated PBS Lite (pH 7.4) containing 5 mM *m*-PD monomer, by cycling the potential between +0.25 and +0.75 V vs. Ag/AgCl at a scan rate of 50 mV s^−1^ for 50 cycles.

The selectivity of the pMEA towards O_2_ and H_2_O_2_ was evaluated by amperometry in the presence of ascorbic acid (AA) and dopamine (DA). Selectivity tests were performed in 40 mL of 0.05 M PBS Lite (pH 7.4) at room temperature (20 °C) with continuous stirring at 240 rpm, at both detection potentials (−0.6 V and +0.7 V vs. Ag/AgCl, respectively). Once a stable baseline was established, the target analyte (O_2_ or H_2_O_2_) were sequentially added to a known concentration of AA (100 µM) and DA (5 µM), and the current response was recorded. Selectivity evaluation (*n* = 4) was performed on 4 ganged recording sites of a single pMEA device. The selectivity ratio was calculated as the ratio of the sensitivity to the target analyte over the sensitivity to each interferent species.

### 2.9. Operational Stability and Biofouling Assessment

To evaluate electrode operational stability and anti-fouling performance under a more biologically representative matrix, a single pMEA device (*n* = 4 ganged recording sites) was initially calibrated for O_2_ and H_2_O_2_ and then continuously polarized at the respective working potentials in an aCSF + glucose + BSA solution for 4 h at room temperature, after which a second calibration was performed under identical conditions. Baseline drift was calculated as the difference between mean baseline currents recorded immediately before the initial (T_0_) and final (T_f_) calibrations, divided by the elapsed time.

### 2.10. Data Analysis

Data analysis was conducted using MultiTrace v. 4.2, OriginPro 2016 (OriginLab, Northampton, MA, USA) and GraphPad Prism 8 (GraphPad Software, San Diego, CA, USA). The sensitivity of the pMEA to O_2_ and H_2_O_2_ was determined by linear regression analysis over the ranges of 0–50 µM and 0–39.2 µM, respectively. The limit of detection (LOD) was defined as the concentration corresponding to a signal-to-noise ratio of 3. Response times (t_90_–t_10%_) were determined by fitting the amperometric current traces to a Boltzmann sigmoid function by calculating the time to reach 10% and 90% of the steady-state response. Values are given as the mean ± standard deviation (SD).

## 3. Results and Discussion

### 3.1. Morphology and Chemical Analysis

All pMEAs used in this work were ganged 64-channel arrays with eight recording sites, including two recording-site regions on each electrode shank, which were designed for (sub)cortical recording, as highlighted in the dashed blue rectangles in Figure 1B. The 64 channels were evenly distributed across 4 shanks each approximately 5.4 mm long, in a boot-lace layout (Figure 1A,B). Each active site had an estimated geometric area of 7.06 × 10^−6^ cm^2^ (30 µm diameter).

To characterize the elemental composition of the pMEA active surface, EDS was employed. Since each element has distinct energy levels, the emitted X-rays can be used to identify the elements present in the sample. As shown in Figure 1C, the active surface primarily consists of Au and Pt (approximately 64.3% and 31.8% respectively), with smaller amounts of C and O. The high Au content observed in the EDS spectrum can be attributed to the penetration depth of the electron beam at 10 keV, which extends beyond the thin outermost Pt layer (25 nm), resulting in a high Au signal relative to the true composition of the outermost surface composition. Because EDS is not intrinsically surface-sensitive, the Au/Pt proportions measured at 10 keV reflect the elemental composition of the multilayer stack within the electron beam interaction volume rather than exclusively the outermost surface. The characteristic hydrogen adsorption/desorption and Pt oxide formation/reduction features observed by cyclic voltammetry (Section 3.2) indicate that the electrochemically accessible surface is predominantly Pt. Moreover, no resolvable Au oxide reduction peak was detected. Nevertheless, trace or highly localized Au exposure cannot be completely excluded. The outermost Pt layer constitutes the electrochemically accessible surface, as confirmed by the characteristic Pt behavior observed in cyclic voltammetry in both acidic and neutral media (Section 3.2) [13,30].

To further examine the surface morphology, SEM micrographs were acquired. As shown in Figure 1D, the planar surface of the multilayer pMEA displays a uniform surface, with no visible micro-cracks or defects, suggesting a high degree of precision in the fabrication. Higher-magnification images (Figure 1E) revealed well-defined, fine-grained structures characteristic of the e-beam evaporation process used to deposit the stack metal layer. This uniformly distributed nanoscale roughness, arising from the deposition process, is expected to support excellent electrical conductivity and mechanical stability, both of which are critical for the pMEA performance [31]. Figure 1F shows a cross-sectional SEM micrograph revealing a deposited metal layer approximately 200 nm thick, in agreement with the manufacturing specifications of the pMEA [32].

**Figure 2:**
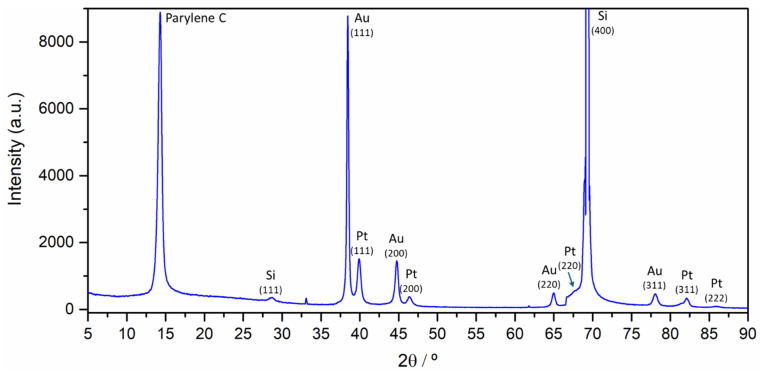
X-ray diffraction spectrum of the pMEA Ti/Pt/Au/Pt metal stack deposited on a silicon carrier wafer, with identification of the principal diffraction peaks corresponding to Parylene C, platinum (Pt), gold (Au), and silicon (Si). Spectra were acquired at 40 kV and 15 mA using CuKα radiation.

To further characterize the pMEA, XRD was used to investigate the atomic and molecular structure of the silicon carrier wafer with the Ti/Pt/Au/Pt metal stack [33]. As illustrated in Figure 2, the positions and intensities of the diffraction peaks in the XRD spectra reveal the presence of multiple crystalline phases, consistent with a layered material structure. Each diffraction peak corresponds to a specific set of lattice planes in the crystal structure. The peak at approximately 2θ ≈ 13° is a low-angle feature likely associated with an organic or polymeric structure, such as Parylene C [34]. Sharp and intense peaks at 2θ ≈ 28° and 69° are attributed to crystalline silicon phases (Si (111) and Si (400), respectively) [33]. Peaks at 2θ ≈ 40°, 47°, 68°, and 82° correspond to Pt (111), Pt (200), Pt (220), and Pt (222), confirming a Pt face-centred cubic (FCC) structure [35,36]. Similarly, peaks at 2θ ≈ 38°, 44°, and 64° are assigned to Au (111), Au (200), and Au (220), characteristic of Au’s FCC structure [35,37].

The Si (400) peak exhibits the highest intensity, reflecting the dominance of the crystalline silicon in carrier wafer. The Au and Pt peaks are moderately intense, consistent with the thin-film nature of these layers and their expected quantities in the pMEA stack.

### 3.2. Electrochemical Behavior in Acidic Electrolyte and in Neutral PBS

Characterization of the electrochemical behavior of Pt pMEAs in acidic media is important for understanding their catalytic properties, particularly in reactions such as the hydrogen evolution reaction (HER) and oxygen reduction reaction (ORR). Acidic solutions significantly enhance the kinetics, leading to more efficient electron transfer processes [30,38].

**Figure 3:**
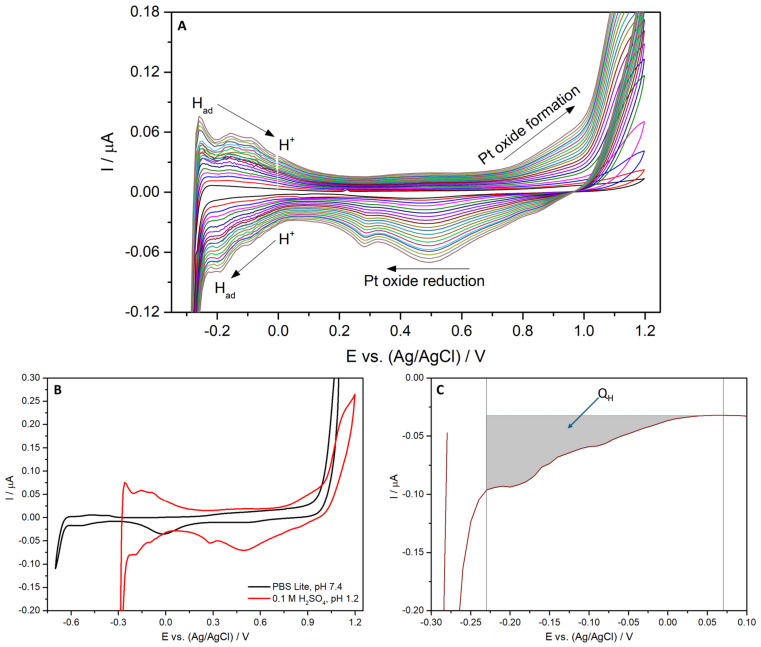
Electrochemical behavior in acidic and neutral electrolyte media. (**A**) Successive cyclic voltammograms (10th scan) recorded at increasing scan rates (50–1000 mV s^−1^) in N_2_-saturated 0.1 M H_2_SO_4_, illustrating typical Pt oxide formation and reduction, hydrogen adsorption (two peaks) and desorption (three peaks), as well as double-layer regions. (**B**) Cyclic voltammograms (200 mV s^−1^) recorded in N_2_-saturated 0.05 M PBS Lite (pH 7.4, black line) and N_2_-saturated 0.1 M H_2_SO_4_ (pH 1.2, red line), demonstrating the positive shift in hydrogen evolution potential and increased currents for Pt oxide formation and reduction at lower pH on the Pt surface of the pMEAs. (**C**) Representative cyclic voltammogram recorded in N_2_-saturated 0.1 M H_2_SO_4_ for the determination of the ECSA of the Pt ganged sites on the pMEA. The shaded area (Q_H_) corresponds to the charge associated with hydrogen adsorption, integrated between E_1_ = −0.23 V and E_2_ = +0.07 V vs. Ag/AgCl (vertical lines), which was used to calculate the ECSA.

Figure 3A shows cyclic voltammograms recorded for a single ganged site of the pMEA between −0.3 and +1.2 V vs. Ag/AgCl at scan rates ranging from 50 to 1000 mV s^−1^. The voltammograms exhibit the characteristic features of a Pt surface, including hydrogen adsorption peaks at −0.05 and −0.19 V vs. Ag/AgCl during the cathodic sweep and three hydrogen desorption peaks at −0.26, −0.15 and −0.09 V vs. Ag/AgCl during the anodic sweep [30,39]. The presence of multiple desorption peaks suggests surface roughness or the presence of different adsorption sites, reflecting the interactions between hydrogen and the Pt surface that underpin various electrochemical processes [30]. Distinct anodic and cathodic peaks corresponding to the formation and reduction of Pt oxides are also observed, namely an oxidation wave at E > 0.5 V corresponding to the formation of Pt-O and Pt-OH oxide species and a reduction peak at E ≈ −0.5 V corresponding to their reduction. These redox features provide important insights into the surface chemistry and catalytic properties of the electrode [40].

Figure 3B compares voltammograms recorded in neutral media between −0.7 and +1.1 V vs. Ag/AgCl and in acidic media between −0.3 and +1.2 V vs. Ag/AgCl. In acidic electrolyte, the higher proton availability leads to more pronounced hydrogen adsorption and desorption peaks reflecting enhanced electrocatalytic activity. By contrast, in neutral media, the lower proton concentration suppresses these hydrogen adsorption features, resulting in less distinct peaks and potentially lower overall catalytic activity compared to the acidic conditions [30]. As shown in Figure 3B, the potential window in neutral media spans approximately 1.5 V compared to approximately 1.0 V in acidic media, consistent with the reduced occurrence of side reactions at neutral pH [30,41].

### 3.3. Electrochemical Active Surface Area

Cyclic voltammetry in acidic media can also provide important information regarding the electrochemical active surface area (ECSA) of the Pt electrode. The hydrogen adsorption peaks observed at negative potentials are indicative of hydrogen underpotential deposition (H_UPD_) on the Pt surface [13], and the area under these peaks is proportional to the number of active sites available [30,42]. Because ECSA is closely related to catalytic performance, it represents a key parameter for evaluating Pt electrodes in electrochemical applications [43].

As shown in Figure 3C, the hydrogen adsorption charge (Q_H_) was determined by integrating the current in the hydrogen adsorption region of the negative-going sweep of the CV recorded in 0.1 M H_2_SO_4_, after correction for the double-layer pseudocapacitance. The integration was performed between E_1_ = −0.23 V and E_2_ = +0.07 V, corresponding to the onset and end of the hydrogen adsorption region, respectively, as defined by the voltammogram (Figure 3C). Q_H_ was calculated according to Equation (1):

(1) $$Q_{H}=\int_{E_{1}}^{E_{2}}\frac{I\left(E\right)}{\upsilon }dE$$

which expresses the relationship between electric current and charge in an electrochemical system, where (Q_H_) is the total electric charge that is passed through the system during a specific process, E_1_ and E_2_ are the integration limits of the hydrogen adsorption region, and *υ* is the scan rate. The ECSA was then calculated by dividing the integrated charge by the established hydrogen charge density for Pt of 210 µC cm^−2^ [30,39,44], leading to Equation (2):

(2) $$ECSA=\frac{Q_{H}}{210\;\mu C\;{cm}^{-2}}$$

The ganged active site of the pMEA exhibited an ECSA of 3.52 × 10^−5^ ± 0.58 ×10^−5^ cm^2^.

### 3.4. Electrochemical Impedance Spectroscopy

**Figure 4:**
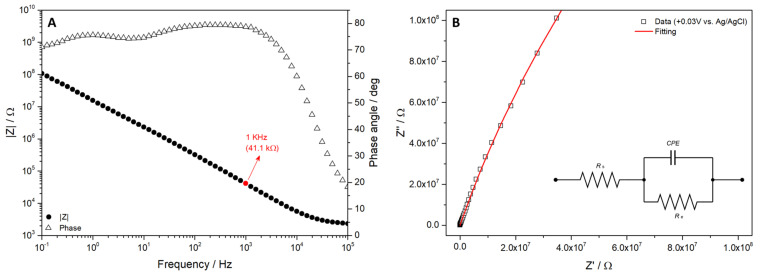
EIS analysis. (**A**) Bode plot showing the impedance magnitude |Z| (filled circles, left axis) and phase angle (open triangles, right axis) as a function of frequency for a Pt ganged site of the pMEA, recorded at OCP (+0.03 V vs. Ag/AgCl). The red-filled circle highlights the |Z| value at 1 kHz (41.1 kΩ). (**B**) Nyquist plot of the experimental EIS data (open squares) for the Pt ganged site. The red line represents the fitted model using the equivalent circuit shown in the inset, comprising R_s_ (solution resistance) in series with a parallel combination of CPE (constant phase element) and a R_e_ (leakage resistance).

Electrochemical impedance spectroscopy (EIS) was used to investigate the interfacial and electrical properties of the Pt pMEA, providing insight into the electrode–electrolyte interface. The resulting Bode and Nyquist plots are shown in Figure 4A,B, respectively. The impedance data was fitted to the equivalent circuit described in Section 2.7 (inset, Figure 4B), comprising an R_s_ in series with a parallel CPE and R_e_. This circuit topology captures the essential features of an electrode interface, where double-layer charging dominates the response across the entire frequency window and no significant faradaic process is present [45].

The fitted parameters are listed in Table 1. The R_s_ value of 2.48 kΩ reflects the ohmic resistance of the PBS electrolyte. The CPE describes the non-ideal double-layer capacitance of the Pt surface, with an exponent value of *n* = 0.84. The deviation of *n* from unity reflects the distributed capacitive response arising from nanoscale surface roughness introduced during e-beam evaporation of the Pt layer, consistent with the surface morphology observed by SEM [46]. The leakage resistance R_e_ converged to a value of 1.3 GΩ, several orders of magnitude larger than the maximum measured impedance, confirming that the electrode behaves as a near-ideal blocking interface in PBS at OCP with no redox-active species present.

In EIS studies, the impedance at 1 kHz is commonly used as a standard reference frequency in neural electrode characterisation, as it aligns with the characteristic range of bioelectrical signals and the impedance of biological tissues [47,48]. At this frequency, the impedance is governed primarily by the double-layer capacitance rather than by charge transfer resistance [45]. The ganged site of the pMEA exhibited a total impedance of 41.1 kΩ (1.17 Ω cm^2^) at 1 kHz, a value which falls within the range considered suitable for in vivo neural recording and is directly associated with an improved signal-to-noise ratio [39,47]. These low impedance values, combined with the favourable surface characteristics revealed by CV, further support the use of pMEAs for concurrent electrophysiological and neurochemical monitoring [7].

### 3.5. Oxygen Reduction Reaction at the Pt Surface

**Figure 5:**
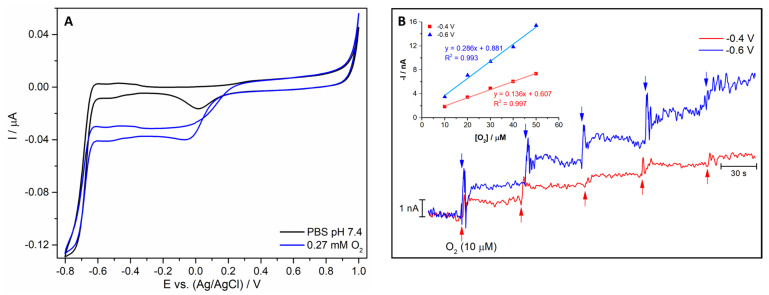
Electrochemical behavior of oxygen reduction on the ganged Pt site of the pMEA. (**A**) Cyclic voltammograms recorded at 100 mV s^−1^ in 0.05 M PBS Lite (pH 7.4), comparing the absence (N_2_-saturated, black line) and the presence (0.27 mM O_2_, blue line) of oxygen. (**B**) Representative amperometric calibration of a single ganged site at −0.4 V (red) and −0.6 V (blue) vs. Ag/AgCl, showing five sequential additions of O_2_ (10 µM each; concentration range 0–50 µM). The corresponding calibration curves for each applied potential are shown in the inset. Arrows indicate the time points of each addition.

Pt is a well-established electrocatalyst for the oxygen reduction reaction (ORR), producing a faradaic reduction current proportional to O_2_ concentration that forms the basis of amperometric O_2_ sensing [39,49,50]. Figure 5A shows representative cyclic voltammograms recorded in PBS (pH 7.4) under N_2_-saturated and air-saturated (0.27 mM O_2_) conditions. In the presence of O_2_ (blue curve), the reduction current begins to increase at approximately −0.2 V, indicating the onset of the ORR, and reaches a plateau ca. −0.4 V, suggesting that this potential is favourable for detecting O_2_ with minimal interference. By contrast, the O_2_-free CV (black curve) exhibits minimal current between −0.2 V and −0.6 V, indicating low background interference in this region. At more negative potentials (ca. −0.6 V vs. Ag/AgCl), the onset of HER is evident from the sharp increase in current observed in both curves. Based on these observations, the optimal working potential for O_2_ monitoring lies between −0.4 V and −0.6 V, where the ORR plateau region is established and interference from HER and capacitive currents remains limited.

Figure 5B shows a representative calibration of a ganged site of the pMEA at −0.4 V and −0.6 V vs. Ag/AgCl, with the corresponding calibration curves (inset). At −0.4 V, the ganged Pt site exhibited an O_2_ sensitivity of −0.13 ± 0.01 nA μM^−1^ (*n* = 8; R^2^ = 0.995 ± 0.003), corresponding to an area-normalised sensitivity of −6.51 ± 2.47 mA mM^−1^ cm^−2^ and an LOD of 5.5 ± 2.1 nM. At −0.6 V, the sensitivity increased to −0.25 ± 0.04 nA μM^−1^ (*n* = 8; R^2^ = 0.996 ± 0.002), with a normalised sensitivity of −11.54 ± 4.75 mA mM^−1^ cm^−2^ and an LOD of 5.4 ± 1.4 nM. A rapid response is observed for each O_2_ addition, with a response time (t_90_–t_10%_) of 5.7 ± 0.6 s (*n* = 8, R^2^ = 0.981 ± 0.009). The amperometric response remained linear up to 150 µM (R^2^ = 0.993 ± 0.002). Further extension of the linear range was constrained by back-diffusion of O_2_ into the deoxygenated calibration solution and by the increasing dilution effect of the injected calibration volumes at higher analyte concentrations. However, this concentration range is higher than the O_2_ concentration levels expected to be found in vivo in tissues [51]. At more negative potentials, the greater overpotential for the ORR enhances the reduction kinetics, resulting in a higher faradaic current. This relationship provides a practical means of tuning the electrode response to the concentration range of relevant species for a given application. In view of the higher sensitivity achieved at −0.6 V together with a comparable LOD and a rapid response time, this potential was selected as the optimal working potential for O_2_ monitoring in agreement with previous reports [13,52].

The sensitivity and LOD values for O_2_ detection using microelectrodes typically vary due to factors such as surface area, electrode geometry, and the electrochemical techniques employed. The sensitivity obtained in this study is consistent with that reported for other Pt and carbon-based microelectrodes [13,53,54,55,56]. Moreover, the LOD is lower than those reported for other microelectrode designs [13,53,56,57], underscoring the suitability of the present pMEA for monitoring O_2_ fluctuations in brain tissue, where basal O_2_ levels are approximately 30 μM [13,58]. Table 2 summarizes the key analytical performance metrics of the present pMEA alongside recently reported O_2_-sensing microelectrodes, highlighting their favorable sensitivity, LOD, and response time relative to comparable devices.

### 3.6. Hydrogen Peroxide Oxidation Reaction at the Pt Surface

The electrochemical oxidation of H_2_O_2_ on a Pt surface is of particular interest in oxidase-based biosensors (e.g., glucose or lactate biosensors) [5,61,62]. The reaction begins with the adsorption of H_2_O_2_ onto the Pt surface, which facilitates subsequent electrochemical oxidation. Upon application of a sufficiently positive potential, the Pt surface promotes the transfer of 2 electrons from the H_2_O_2_ to the electrode, resulting in the oxidation of H_2_O_2_ into O_2_ and protons. During this process, intermediate species may form, contributing to the overall reaction mechanism. The oxidation products, O_2_ and protons, are then released from the electrode surface, thereby generating active sites for further reactions. The overall reaction is described by reaction 3 [63]:

(3) $$H_{2}O_{2}\to O_{2}+2H^{+}+2e^{-}$$

Several factors influence the efficiency of H_2_O_2_ oxidation on a Pt surface. Solution pH plays a role, as the reaction proceeds more efficiently in acidic or neutral conditions where protons are available to facilitate the desorption step. The electrode surface area and roughness are also important, since a larger electroactive surface area and a greater nanoscale roughness increase the number of available active sites for H_2_O_2_ adsorption and intermediate reactions. In contrast, contaminants or impurities present on the Pt surface may reduce the electrode performance by blocking active sites or modifying the surface properties [64].

**Figure 6:**
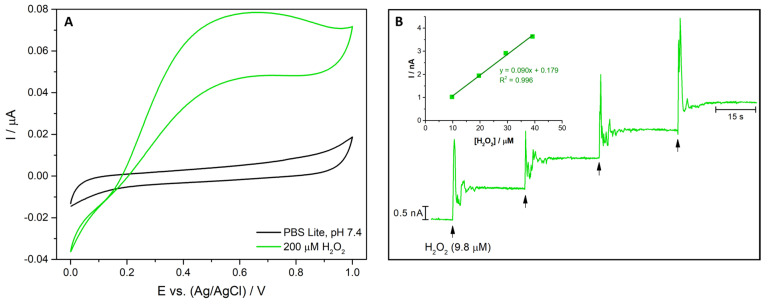
Electrochemical behavior of hydrogen peroxide oxidation on the ganged Pt site of the pMEA. (**A**) Cyclic voltammograms recorded at 100 mV s^−1^ in N_2_-saturated 0.05 M PBS Lite (pH 7.4), comparing the absence (black line) and the presence (green line) of 200 µM H_2_O_2_. (**B**) Representative amperometric calibration of a single ganged site at +0.7 V vs. Ag/AgCl showing four sequential additions of H_2_O_2_ (9.8 µM each; concentration range 0–39.2 µM), with the corresponding calibration curve shown in the inset. Arrows indicate the time points of each addition.

Figure 6A shows representative cyclic voltammograms recorded in PBS (pH 7.4) in the absence and presence of 200 µM of H_2_O_2_. In the presence of H_2_O_2_ (green curve), the oxidation current increases progressively from +0.2 V, reaching a broad maximum around +0.6–0.7 V, beyond which no further increase is observed. By contrast, the H_2_O_2_-free scan (black curve) shows minimal current throughout this range, confirming low background interference. Based on these observations, +0.7 V vs. Ag/AgCl was selected as optimal working potential, as it provides a stable oxidation current, while minimizing noise.

Figure 6B shows a representative calibration of a ganged pMEA site at +0.7 V vs. Ag/AgCl, and the corresponding calibration curve (inset). A rapid response was observed for each H_2_O_2_ addition, with a response time (t_90_–t_10%_) of 8.5 ± 0.2 s (*n* = 8, R^2^ = 0.994 ± 0.001). The ganged Pt site exhibited a H_2_O_2_ sensitivity of 88.13 ± 7.61 nA mM^−1^ (*n* = 8, R^2^ = 0.996 ± 0.002), an area-normalised sensitivity of 2.56 ± 0.48 mA mM^−1^ cm^−2^, and an LOD of 41.9 ± 5.6 nM. The amperometric response remained linear up to 2 mM (*n* = 4, R^2^ = 0.998± 0.002), confirming that the linear dynamic range extends beyond the physiologically relevant concentrations.

As noted above, both sensitivity and LOD are highly influenced by multiple factors, including electrode geometry, surface properties, and experimental conditions. The sensitivity obtained is particularly higher than that reported for other Pt-based microelectrodes [61,65,66], and significantly higher than values reported for modified glassy carbon electrode [67,68,69], reflecting the favourable electrocatalytic properties of the pMEA Pt surface towards H_2_O_2_ oxidation. The LOD falls within the range reported for microelectrode-based H_2_O_2_ detection (10 nM to 10 µM) [70] and is well suited for monitoring H_2_O_2_ in biological media where concentrations are typically in the low micromolar range. A comparison with other Pt-based microelectrode arrays is presented in Table 3. These results support the suitability of these pMEAs for monitoring H_2_O_2_ in brain extracellular space, including studies of oxidative stress in neurological disorders [71,72].

### 3.7. Selectivity Evaluation

To assess the selectivity of the pMEA against common electroactive interferents present in the brain extracellular environment, the responses of both bare Pt and *m*-PD-coated sites to AA and DA were evaluated by amperometry at both detection potentials. *m*-PD is a well-established permselective coating for Pt microelectrodes in neurochemical biosensing, acting as a size exclusion barrier that restricts the access of larger electroactive molecules to the electrode surface while allowing the smaller H_2_O_2_ molecule to diffuse through freely [65,77,78,79]. AA and DA were selected because of their electroactive nature and their presence in the brain extracellular space [71,80,81,82].

**Figure 7:**
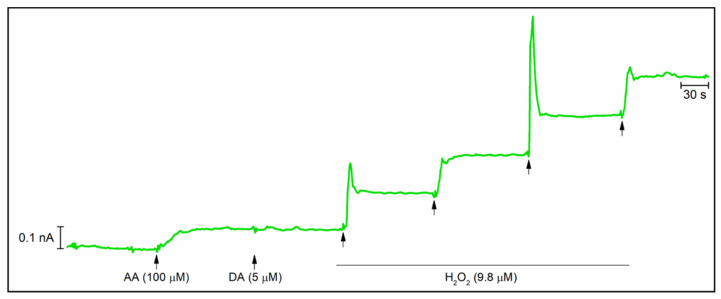
Representative recording of the *m*-PD-coated pMEA ganged site at +0.7 V vs. Ag/AgCl, showing the current response to sequential additions of ascorbic acid (AA, 100 µM), dopamine (DA, 5 µM) and H_2_O_2_ (9.8 µM) in 0.05 M PBS Lite (pH 7.4). Arrows indicate the time points of each addition.

Selectivity at +0.7 V vs. Ag/AgCl was initially evaluated at the bare Pt electrode with sequential additions of AA (100 µM), DA (5 µM) and H_2_O_2_ (9.8 µM). This resulted in current changes corresponding to H_2_O_2_–interferent selectivity ratios, on a molar basis, of 1.2 ± 0.06:1 (*n* = 4) for AA and 0.85 ± 0.35:1 (*n* = 4) for DA. Following *m*-PD electrodeposition, selectivity towards AA and DA was increased to 20.3 ± 0.63:1 (*n* = 4) and 76.9 ± 9.89:1 (*n* = 4), respectively, as shown in Figure 7. The corresponding blocking efficiencies were 95% to AA and 99% to DA, demonstrating effective exclusion of both interferents by the permselective membrane. At −0.6 V vs. Ag/AgCl, the bare Pt electrode already exhibited negligible interference from AA and DA with selectivity ratios of 306.3 ± 51.6:1 (*n* = 4) and 63.6 ± 4.60:1 (*n* = 4), respectively. Following *m*-PD coating, selectivity values remained comparably high, reaching 532.8 ± 23.4:1 (*n* = 4) for AA and 88.0 ± 7.39:1 (*n* = 4) for DA. These values corresponded to blocking efficiencies of 99.8% to AA and 98.8% to DA.

Taken together, these results demonstrate that the pMEA Pt surface exhibits inherent selectivity for O_2_ detection at cathodic potentials, and that *m*-PD electrodeposition effectively suppresses interferent signals at oxidative potentials without compromising analytical performance.

### 3.8. Operational Stability and Biofouling Effects

To assess the operational stability and biofouling performance of the pMEA under more physiologically challenging conditions, calibrations were performed before (T_0_) and after (T_f_) a 4 h continuous polarization period in artificial cerebrospinal fluid supplemented with glucose and BSA. For O_2_, baseline current drifted at a rate of −0.34 ± 0.10 nA h^−1^ (−1.8 ± 0.5% h^−1^), with a sensitivity and LOD decrease of 9.6 ± 0.4% and a 22.7 ± 5.5% over the 4 h period, respectively. For H_2_O_2_, baseline current drifted at a rate of −0.005 ± 0.002 nA h^−1^ (−14.7 ± 5.7% h^−1^), with a sensitivity and LOD decrease of approximately 36.2 ± 1.3% and 51.4 ± 12.7% respectively, over the 4 h period.

The observed decrease in sensitivity for both analytes is consistent with the formation of a diffusional fouling layer on the Pt surface, arising from the adsorption of proteinaceous components (BSA) present in the aCSF, consistent with fouling mechanisms broadly reported for carbon- and metal-based neurochemical microelectrodes [83,84]. Such a layer would increase the effective path length for H_2_O_2_ to reach the electroactive Pt surface, thereby slowing the amperometric response. The LOD improved for both analytes following the stabilization period, which can be attributed to a reduction in baseline noise observed in the T_f_ calibration relative to T_0_.

These results indicate that, while decrease in sensitivity occurs over a 4 h exposure to a biologically representative matrix, the pMEA retains functional responsiveness to both O_2_ and H_2_O_2_ throughout the tested period.

## 4. Conclusions

This work provides a comprehensive morphological and electrochemical characterization of a Parylene C-based pMEA featuring a Ti/Pt/Au/Pt multilayer active surface. The active sites exhibited a uniform, defect-free surface with nanoscale platinum structures while cyclic voltammetry confirmed typical bulk Pt behavior and diffusion-controlled kinetics for both O_2_ reduction and H_2_O_2_ oxidation. The low impedance at 1 kHz further demonstrates the suitability of the platform for in vivo neural recording. The Pt-based pMEAs showed excellent electrocatalytic activity, high sensitivity and a low limit of detection, and rapid response times for both analytes with linear dynamic ranges extending to 150 µM for O_2_ and 2 mM for H_2_O_2_, comparing favorably with recently reported Pt-based microelectrode arrays. For O_2_ detection, −0.6 V vs. Ag/AgCl was selected as the optimal working potential, where a well-defined reduction plateau region is established. For H_2_O_2_ detection, +0.7 V vs. Ag/AgCl provided a stable oxidation current with minimal noise. The selectivity evaluation showed that O_2_ detection at negative potentials is unaffected by interference from common electroactive species, whereas for H_2_O_2_ detection, the electropolymerized *m*-phenylenediamine membrane efficiently minimized ascorbic acid and dopamine interference. Operational stability assessments in a biologically representative matrix (aCSF supplemented with glucose and BSA) revealed a decrease in sensitivity for both analytes over 4 h, consistent with the formation of a diffusional fouling layer on the Pt surface, while the pMEA retained functional responsiveness to both O_2_ and H_2_O_2_ throughout the tested period. Overall, these results establish this pMEA platform as a promising neural probe for in vivo multimodal monitoring, enabling the integration of neurochemical sensing with electrophysiological recording. In addition, they provide a robust electrochemical basis for the future development of enzyme-based and other advanced biosensors for real-time in vivo measurements in the brain.

## Figures and Tables

**Table 1:** Summary of fitted parameters results for impedance spectroscopy measurements for the Pt pMEAs.

R_s_ (kΩ)	R_e_	CPE	*n*
2.48	1.3 GΩ	13.0 nF s*^n^*^−1^	0.84
	45.76 ^[a]^ kΩ cm^2^	369.3 ^[a]^ µF cm^−2^ s*^n^*^−1^	

^[a]^ Values normalized by ECSA.

**Table 2:** Analytical Performance Comparison of Microelectrode Arrays for Oxygen Detection ^a^.

Microelectrode Type	Electrode Material (Dimensions, μm)	Sensitivity (nA μM^−1^)	Sensitivity/Unit Area (mA mM^−1^ cm^−2^)	LOD (nM)	|Z| at 1 kHz/Unit Area (Ω cm^2^)	Response Time (s)	Ref.
Flexible (Parylene-C)	Pt (30 ⌀)	−0.25 ± 0.04	−11.54 ± 4.75	5.4 ± 1.4	1.17	5.7 ± 0.6	This work
Flexible (SU-8)	Pt (20 × 20)	−0.00426 ^b^	−1.065 ^b^	nd	nd	<15	[59]
Ceramic	Pt (15 × 333)	−0.16 ± 0.02	−3.2 ± 0.5	330 ± 200	10.8	na	[13]
Silicon	Pt (30 ⌀)	nd	nd	nd	nd	nd	[60]
Silicon	Pt (17.5 × 17.5)	−0.0082	−2.68	20,090	nd	nd	[52]

^a^ Data is presented as mean ± SD; ^b^ Calculated from data in publication; nd, not determined.

**Table 3:** Analytical performance comparison of microelectrode arrays for hydrogen peroxide detection ^a^.

Microelectrode Type	Electrode Material (Dimensions, μm)	Sensitivity (nA μM^−1^)	Sensitivity/Unit Area (mA mM^−1^ cm^−2^)	LOD (nM)	|Z| at 1 kHz/Unit Area (Ω cm^2^)	Response Time (s)	Ref.
Flexible (Parylene-C)	Pt (30 ⌀)	88.13 ± 7.61	2.56 ± 0.48	41.9 ± 5.6	1.17	8.5 ± 0.2	This work
Ceramic	Pt (50 × 50)	0.0122	0.488 ^b^	133	nd	nd	[73]
Ceramic	Pt (100 × 50)	0.006743	0.135 ^b^	nd	nd	nd	[74]
Silicon	Pt Black (20 ⌀)	nd	nd	10	nd	<1	[75]
Glass	Pt Black (20 ⌀)	0.0172 ^b^	5.48	nd	nd	nd	[76]

^a^ Data is presented as mean ± SD; ^b^ Calculated from data in publication; nd, not determined.

## Data Availability

The raw data supporting the conclusions of this article will be made available by the authors on request.
